# Evaluation of Cancer Care After Medicaid Expansion Under the Affordable Care Act

**DOI:** 10.1001/jamanetworkopen.2020.17544

**Published:** 2020-09-29

**Authors:** Michelle C. Salazar, Maureen E. Canavan, Samantha L. Walters, Jeph Herrin, Jason L. Schwartz, Michael Leapman, Daniel J. Boffa

**Affiliations:** 1 Section of Thoracic Surgery, Department of Surgery, Yale University School of Medicine, New Haven, Connecticut; 2 National Clinician Scholars Program, Yale University School of Medicine, New Haven, Connecticut; 3 Cancer Outcomes Public Policy and Effectiveness Research (COPPER) Center, Department of Internal Medicine, Yale University School of Medicine, New Haven, Connecticut; 4 Section of Cardiovascular Medicine, Department of Internal Medicine, Yale University School of Medicine, New Haven, Connecticut; 5 Department of Health Policy and Management, Yale School of Public Health, New Haven, Connecticut; 6 Department of Urology, Yale University School of Medicine, New Haven, Connecticut

## Abstract

This cohort study evaluates different approaches for estimating the population of individuals eligible for Medicaid expansion under the Affordable Care Act in the context of researching changes in cancer care after Medicaid expansion.

## Introduction

By 2017, more than 17 million previously uninsured people in the US had gained insurance through Medicaid expansion (ME), which was instituted as part of the Affordable Care Act of 2010.^1^ Given prior associations between insurance status and cancer outcomes, there has been growing interest in the impact of ME on the 1.7 million people diagnosed with cancer annually in the US.^2^ However, individuals more likely to benefit from ME would have been those previously uninsured who gained insurance by meeting eligibility criteria for ME, an estimated 76% of uninsured patients.^3^ Focusing on expansion-eligible patients for research can be challenging because most cancer databases lack data on key eligibility criteria for expansion (income ≤138% of the federal poverty level and the absence of other insurance coverage opportunities).

Attempts to understand the impact of ME on cancer prevalence and care have approached study of the expansion-eligible population differently. Some have studied the entire cancer population,^4^ whereas others have studied cohorts of patients likely to be ME eligible (eg, those with low incomes or in racial/ethnic minority groups).^5^ Different approaches have resulted in varying proportions of expansion-eligible patients within study populations. Using the prevalence of stage I breast cancer diagnosis as an example of an outcome potentially impacted by access to insurance (ie, more insurance equals more screening and, consequently, earlier cancer diagnosis and care), we evaluated 3 previously described approaches and 2 novel approaches to focus on expansion-eligible patients.

## Methods

This retrospective cohort study used the American College of Surgeons’ National Cancer Database 2016 breast cancer Participant User File to search for female patients aged 40 to 64 years with newly diagnosed breast cancer. Stage I breast cancer rates in patients from the 19 states that expanded in January 2014 were compared with those from the 19 states that had not expanded by 2016. This study was approved by the Yale School of Medicine review board. Consent was waived because publicly available data were used. The Strengthening the Reporting of Observational Studies in Epidemiology (STROBE) reporting guideline was used.

Five approaches to focusing on expansion-eligible patients were evaluated (Table); 3 of these have been previously reported.^4,5^ The remaining 2 approaches have not previously been described, 1 restricting the cohort to Medicaid and uninsured patients before and after expansion, the other using propensity score matching to match Medicaid patients after expansion with uninsured patients before expansion (see the Table). The primary outcome was the proportion of newly diagnosed female patients who had clinical stage I breast cancer. Difference-in-difference (DiD) modeling comparing expansion and nonexpansion states was used to evaluate the outcome.^4,5^ The parallel trends assumption was tested both graphically and through a linear regression, including an interaction between time and expansion status during the preexpansion period (Figure). The interaction effect was not significant for all methods except for the “all patients” approach. Statistical significance was set at *P* < .05, and all tests were 2-tailed. Analyses were performed using SAS version 9.4 (SAS Institute Inc) and STATA version 16 (StataCorp LLC).

**Table.** Characteristics of the Different Focus Approaches Evaluated^a^^,^^b^

	All patients (n = 231 938)	Likely to be expansion eligible		Medicaid and uninsured patients (n = 29 657)	Matched (n = 9036)^c^^,^^d^
		Low income (n = 39 881)	Race/ethnicity (n = 58 852)		
Approaches reported by	Takvorian et al^4^ and Jemal et al^5^	Crocker et al^6^	Crocker et al^6^	Present study	Present study
Patient population	All patients before and after expansion	All patients residing in lowest quartile of income zip codes	All non-White or Hispanic patients	All patients who had Medicaid or were uninsured before and after expansion	Propensity scores used to identify preexpansion uninsured patients who were similar to postexpansion Medicaid patients
Distribution of insurance status in the preexpansion and postexpansion eras (nonexpansion and expansion states combined), %					
Uninsured					
Preexpansion	3.9	6.7	8.0	29.7	100
Postexpansion	2.9	5.2	6.4	24.4	NA
Medicaid					
Preexpansion	9.3	17.7	16.8	70.3	NA
Postexpansion	9.1	17.2	15.9	75.6	100
Private					
Preexpansion	75.8	60.2	61.4	NA	NA
Postexpansion	76.8	62.0	64.2	NA	NA
Other^e^					
Preexpansion	11.0	15.3	13.8	NA	NA
Postexpansion	11.2	15.6	13.6	NA	NA
Advantages to the approach	All expansion-eligible patients are captured	Should concentrate expansion-eligible patients	Should concentrate expansion-eligible patients	Captures the vast majority of expansion-eligible people, concentrates expansion-eligible patients, and captures expansion-eligible patients who did not obtain Medicaid	Should provide the highest concentration of expansion-eligible patients in the study population before and after expansion
Limitations of the approach	Expansion-eligible patients represent a small minority of patients (around 1.9% of the study population)^f^	Most patients in poor zip codes are not expansion eligible because they were already eligible for Medicaid before expansion	Only focuses on members of ethnic/racial minority groups and therefore does not capture other eligible individuals	Only 14.8% of cohort would be expansion eligible^f^	Will not capture expansion-eligible patients who did not obtain Medicaid and is less likely to capture expansion-eligible patients who are unusual (eg, have atypical sociodemographics)

Abbreviation: NA, not applicable.

^a^ The *Medicaid expansion status state group* field (only available for patients 40 years or older) was used to identify patients who resided in nonexpansion states and January 2014 expansion states. Patients in early or late expansion states were excluded.

^b^ Only patients with complete clinical stage information were included in all analyses.

^c^ Medicaid patients post-expansion were propensity matched to pre-expansion uninsured patients. A 1:1 nearest neighbor propensity match on age, race, Hispanic origin, Charlson-Deyo score, income, and area of residence was used. All post-match standardized differences were found to be <0.1.

^d^ Missing values for baseline covariates were less than 5% and were coded as a separate category for purposes of the propensity score matching. The only exception was income, for which missing values were ultimately excluded because the frequency of missing values was so low that it was impacting the balance of the match.

^e^ Includes Medicare, other government insurance, and unknown insurance status.

^f^ Based on patients living in January 2014 expansion states only.

**Figure.**
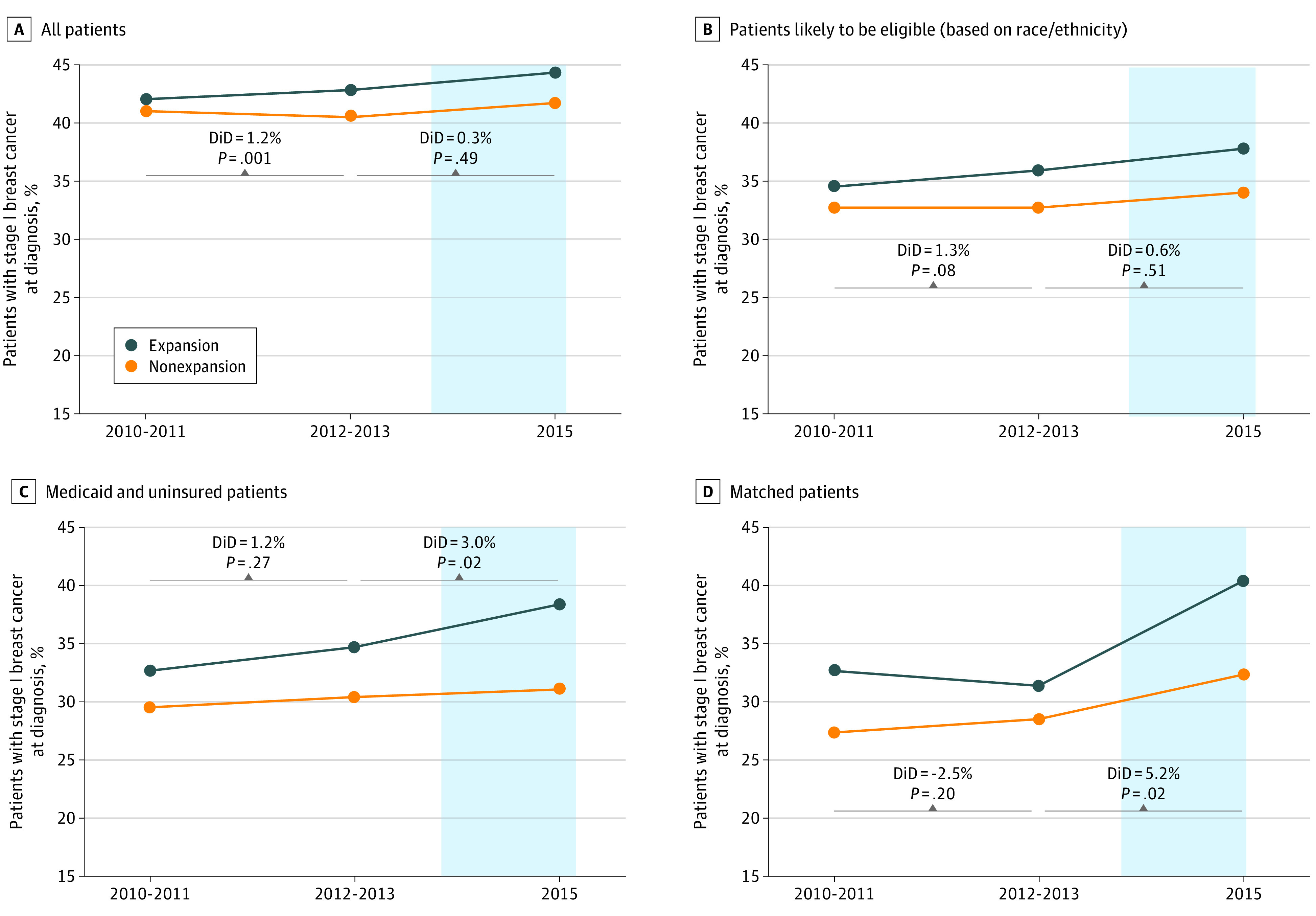
Comparison of Different Approaches to Evaluate Percentages of Stage I Breast Cancer at Diagnosis Before and After Medicaid Expansion in States With and Without Expansion Using Difference-in-Difference (DiD) Modeling Percentages of patients in nonexpansion states and the January 2014 expansion-state groups were measured at 3 time points according to year of diagnosis: 2010-2011 (well before expansion), 2012-2013 (just before expansion), and 2015 (after expansion). The 2010-2011 group was only used to evaluate trends before ME. Year 2014 was excluded as a phase-in period. The blue section indicates the period from the beginning of expansion and after. The graph showing the group who were likely to be expansion eligible as a result of low income is available upon request.

## Results

In this cohort study of different approaches to focus on the population of patients with breast cancer who could have been expansion eligible, the study populations varied in size, from 231 938 (all patients) to 9036 (propensity score matched). The proportions of uninsured and Medicaid patients varied by cohort, ranging from 3.6% and 9.2% for all patients to 50% and 50% for matched patients (Table).

The percentage of the study population (women age 40 to 64 years) who had newly diagnosed stage I breast cancer was tracked at 3 time points: well before expansion (2010-2011), just before expansion (2012-2013), and after expansion (2015) (Figure A-D). The 2010-2011 group was only used to evaluate trends before ME. Year 2014 was excluded as a phase-in period. The proportion of the population with stage I breast cancer increased to varying degrees after expansion, depending on the approach used, from 42.8% in 2012-2013 to 44.3% in 2015 among all patients in expansion states (Figure, A) and from 31.4% in 2010-2011 to 40.4% among propensity score–matched patients in expansion states (Figure, D). Changes associated with expansion were only significant for Medicaid and uninsured patients (DiD, 3.0%; 95% CI, 0.6%-5.3%; *P* = .02) (Figure, C) and matched patients (DiD, 5.2%; 95% CI, 1.2%-9.2%; *P* = .02) (Figure, D).

## Discussion

This retrospective cohort study suggests that different approaches to focusing on expansion-eligible patients are associated with different impressions of the influence of ME on patients with breast cancer. This phenomenon may not be limited to breast cancer; similar variability was seen for other screening-detectable cancers, including prostate, lung, and colon cancer (data available upon request). In the present study, as the proportion of uninsured and Medicaid patients increased within the approaches, the association of ME with a shift toward stage I diagnosis appeared to strengthen. Propensity score matching, which simultaneously considered a range of predictors for expansion-eligible status, was associated with the largest difference after expansion. This approach combined multiple factors associated with insurance status (unlike stratification approaches using solitary predictors such as race/ethnicity or income), potentially yielding the highest concentration of expansion-eligible patients (albeit with less sensitivity). It is important to note that each approach might have its own limitations; for example, the all-patients approach violated the parallel trends assumption (see Table for details).

It should also be noted that other populations could benefit from ME (eg, patients who were on high-deductible plans who were then eligible to switch to Medicaid), but those who were more likely to benefit from ME were individuals who were previously uninsured and gained insurance through ME.

Although there is no perfect way to focus on expansion-eligible patients to model the impact of ME on cancer care, investigators and their audiences should recognize the potential for variations among approaches. Efforts should be made to identify the approach that best aligns with the intended research question, even though it will likely reflect a trade-off between sensitivity and specificity for the expansion-eligible population.
